# Validation of a non-linear index of heart rate variability to determine aerobic and anaerobic thresholds during incremental cycling exercise in women

**DOI:** 10.1007/s00421-022-05050-x

**Published:** 2022-10-21

**Authors:** Marcelle Schaffarczyk, Bruce Rogers, Rüdiger Reer, Thomas Gronwald

**Affiliations:** 1 Department Sports and Exercise Medicine, Institute of Human Movement Science, University of Hamburg, Hamburg, Germany; 2 Department of Internal Medicine, College of Medicine, University of Central Florida, Boston, USA; 3 Institute of Interdisciplinary Exercise Science and Sports Medicine, MSH Medical School Hamburg, University of Applied Sciences and Medical University, Hamburg, Germany

**Keywords:** Ventilatory threshold, Intensity distribution, HRV, Detrended fluctuation analysis, DFA-alpha1

## Abstract

Studies highlight the usage of non-linear time series analysis of heart rate variability (HRV) using the short-term scaling exponent alpha1 of Detrended Fluctuation Analysis (DFA-alpha1) during exercise to determine aerobic and anaerobic thresholds. The present study aims to further verify this approach in women. Gas exchange and HRV data were collected from 26 female participants with different activity levels. Oxygen uptake (VO_2_) and heart rate (HR) at first (VT1) and second ventilatory thresholds (VT2) were compared with DFA-alpha1-based thresholds 0.75 (HRVT1) and 0.50 (HRVT2). Results: VO_2_ at VT1 and VT2 were 25.2 ml/kg/min (± 2.8) and 31.5 ml/kg/min (± 3.6) compared with 26.5 ml/kg/min (± 4.0) and 31.9 ml/kg/min (± 4.5) for HRVT1 and HRVT2, respectively (ICC_3,1_ = 0.77, 0.84; *r* = 0.81, 0.86, *p* < 0.001). The mean HR at VT1 was 147 bpm (± 15.6) and 167 bpm (± 12.7) for VT2, compared with 152 bpm (± 15.5) and 166 bpm (± 13.2) for HRVT1 and HRVT2, respectively (ICC_3,1_ = 0.87, 0.90; *r* = 0.87, 0.90, *p* < 0.001). Bland–Altman analysis for VT1 vs. HRVT1 showed a mean difference of − 1.3 ml/kg/min (± 2.4; LoA: 3.3, − 6.0 ml/kg/min) for VO_2_ and of − 4.7 bpm (± 7.8; LoA: 10.6, − 20.0 bpm) for HR. VT2 vs. HRVT2 showed a mean difference of − 0.4 ml/kg/min (± 2.3; LoA: 4.1, − 4.9 ml/kg/min) for VO_2_ and 0.5 bpm (± 5.7; LoA: 11.8, − 10.8 bpm) for HR. DFA-alpha1-based thresholds showed good agreement with traditionally used thresholds and could be used as an alternative approach for marking organismic transition zones for intensity distribution in women.

## Introduction

Training zone demarcation is often based on physiological thresholds of blood lactate concentration or gas exchange under monitored laboratory or field conditions (Skinner and Mclellan 1980; Mann et al. 2013). Apart from the complexity and costs of these procedures, it is recognized that depending on the lactate threshold approach used, testing protocol, type of exercise, or the expertise of the gas exchange test interpreter (Yeh et al. 1983; Shimizu et al. 1991; Meyer et al. 1996), dissimilar threshold calculations can result in differing training recommendations (Jamnick et al. 2018). The need for objective, non-invasive and low-cost markers for threshold demarcation is apparent. Heart rate (HR) variability (HRV) measures have been tested for years as a potential monitoring tool, although mostly during resting conditions and with focus on vagal-related HRV indices (Buchheit 2014; Michael et al. 2017). Recently, studies have endeavored to evaluate the accuracy of HRV correlation properties, in particular, the short-term scaling exponent alpha 1 of Detrended Fluctuation Analysis (DFA-alpha1) for delineation of physiological thresholds during endurance exercise (Gronwald et al. 2020b, 2021; Rogers et al. 2021a). This index represents the fractal, self-similar nature of cardiac beat-to-beat intervals. For DFA-alpha1 calculation, the root mean square fluctuation of the integrated and detrended data is measured in observation windows of different sizes. This is done using a logarithmic plot of the data against the size of the window. The resulting slope of the line relating the (log) fluctuation to the (log) window size represents the scaling exponent (Mendonca et al. 2010). At low exercise intensity, DFA-alpha1 values usually are near 1.0 or slightly above, signifying a well correlated, fractal pattern (Gronwald et al. 2020b). As intensity rises, the index will drop past 0.75 near the aerobic threshold (AT) then approach uncorrelated, random patterns represented by values near 0.50 and below at higher work rates (Rogers et al. 2021a, b). These observations are attributable to changes in autonomic nervous system balance, namely the withdrawal of the parasympathetic and enhancement of the sympathetic branch as well as the influence of other non-neural factors during endurance exercise (Persson 1996; White and Raven 2014; Qu et al. 2014; Gronwald et al. 2020b). Moreover, DFA-alpha1 appears to be an index that reflects the overall systemic state of internal load (Gronwald and Hoos 2020; Rogers et al. 2021d). In contrast to other typical HRV indexes such as time domain SDNN (total variability as the standard deviation of all normal RR-intervals) which reaches a nadir at the first ventilatory and lactate thresholds (staying suppressed afterward) (Tulppo et al. 1996; Karapetian et al. 2008), DFA-alpha1 possesses a wide dynamic range spanning all exercise intensity domains (Gronwald et al. 2020b). Furthermore, its dimensionless nature allows application independent of an individual’s fitness status and without the need for prior normalization to blood lactate concentration or gas exchange kinetics (Gronwald et al. 2020b; Rogers et al. 2021d, e). Recent investigations showed agreement in the intensities reached at DFA-alpha1 derived thresholds marked as 0.75 (HRVT1) and 0.50 (HRVT2) with the first (VT1) and second ventilatory threshold (VT2) during an incremental treadmill protocol in recreational male runners (Rogers et al. 2021a, b). However, since an electrocardiogram (ECG) was used for RR interval data recording with artifact level lower than 3%, it remained unclear whether the same results can also be confirmed in the general application with commercial chest straps and divergent error presence. A follow-up study examining the effects of missed beat artifact on both DFA-alpha1 as well as the HTVT1 showed minimal effects at levels below 5% using the Kubios “automatic” correction method. In addition, there was a small but significant degree of bias of the HRVT1 (4 bpm) between ECG and the Polar H7 (Rogers et al. 2021c). In addition, the HRVT1 was shown to strongly agree with that of the VT1 in a male cardiac disease population (Rogers et al. 2021e). What remains unclear is the question of whether the HRVT1 and HRVT2 thresholds agree with that of the VT1 and VT2 in a group of female participants. If the use of the fixed 0.75 and 0.50 DFA-alpha1 values for threshold identification is confirmed in that demographic, widespread application for intensity distribution monitoring could result. In addition to benefits for routine sports laboratory work, this metric could provide real-time information about absolute exercise intensity based on systemic internal load without prior lactate or gas exchange testing with only HRV monitoring. However, a generalization of the applicability, especially due to the few trials for large parts of the population as well as exercise protocols requires further investigation. There is a particular need for data on women as it has been demonstrated that there are significant gender differences in HRV-related cardiac stress responses and these may also be reflected in non-linear metrics (Adjei et al. 2018). The reasons behind this disparity could be attributed to hormonal, neuroanatomical and cognitive differences in the sexes with age being an essential modulator (Ramaekers 1998; Balhara et al. 2012). Since the gender difference in cardiac autonomic function narrows between the age of 40 and 50 with women becoming menopausal during this age range, the female hormone estrogen is suspected of having a significant influence which can furthermore be observed during the menstrual cycle (Ramaekers 1998; Yildirir et al. 2001). Therefore, this study aims to extend the validity of DFA-alpha1-based intensity thresholds to female participants tested in a cycling ramp protocol.

## Materials and methods

### Participants

Thirty-one female volunteers aged 20–59 (32 ± 10 years), were examined. Mean body weight was 68 ± 9 kg and mean height comprised 169 ± 6 cm. The participation recruitment was made by word-of-mouth and via social media advertising. Women of any fitness level without previous medical history, current medications or recent illness were eligible for participation. Information on physical activity as well as menstrual cycle and contraception were recorded (Table 1). Participants were asked to abstain from caffeine, alcohol, tobacco, and vigorous exercise 24 h before testing. Before study enrollment, each participant had to give their informed consent. Ethical approval for the study was obtained by the University of Hamburg, Department of Psychology and Movement Science, Germany (reference no.: 2020_328) and is in line with the principles of the Declaration of Helsinki.

**Table 1:** Anthropometrical data and information on physical activity, menstrual cycle and contraception

Participant	Age (years)	Weight (kg)	Height (cm)	Regular exercise?	Menstrual cycle day on test day	Contraception
1	26	76	170	Yes	9	Contraceptive pill
2	27	70	171	Yes	6	No
3	25	71	165	Yes	2	Copper spiral
4	25	60	168	Yes	5	Hormone spiral
5	35	61	170	Yes	Irregular	No
6	21	69	164	Yes	20	Contraceptive pill
7	27	55	166	No	9	No
8	29	75	159	No	10	No
9	58	59	168	No	Menopause	No
10	29	55	161	Yes	26	Contraceptive pill
11	28	67	170	No	21	No
12	25	89	172	Yes	3	Hormone ring
13	22	60	161	Yes	20	No
14	49	59	159	Yes	Unknown	Unknown
15	30	77	173	Yes	Unknown	Hormone spiral
16	41	76	176	Yes	1	No
17	21	81	187	Yes	1	No
18	20	73	179	Yes	21	No
19	32	55	158	Yes	6	No
20	57	69	171	No	Menopause	No
21	33	74	168	No	27	Hormone ring
22	27	65	171	No	4	No
23	31	64	174	Yes	21	No
24	27	67	176	Yes	Unknown	Hormone spiral
25	38	66	172	Yes	13	No
26	38	66	164	No	18	No
Mean (SD)	32 (± 10)	68 (± 9)	169 (± 6)	−	−	−

*SD*   standard deviation

### Exercise protocol

All tests were carried out in a sports medicine laboratory at a constant room temperature of 20 degrees and a humidity of 40%. Participants performed a ramp protocol on a mechanically braked cycle (Ergoselect 4 SN, Ergoline GmbG, Germany). After 3 min at 50 W, the power increased by 1 W every 3.6 s (equivalent to 16.7 W per min or 50 W every 3 min). Rating of perceived exertion (RPE) was given before start, every 3 min and immediately after stopping. HR, HRV measures and gas exchange kinetics were recorded continuously with a chest strap (H10, Polar Electro Oy, Kempele, Finland; sampling rate: 1000 Hz) and a mobile application (Elite HRV, Version 5.5.1), as well as with a metabolic analyzer (Quark CPET, Omnia software, version 1.6.5, module A-67-100-02, Cosmed, Italy). The protocol was terminated when the participants could not either hold the predetermined cycling cadence (60 rpm) or due to voluntary exhaustion, discomfort or pain. Exhaustion was assumed when the following criteria were fulfilled: (A) heart rate > 90% of the maximum predicted heart rate (prediction model according to Tanaka et al. (2001): 208 − (0.7 × age) and (B) respiratory quotient > 1.1. Maximum oxygen uptake (VO_2max_) was defined as the average VO_2_ over the last 30 s of the test. For maximum HR (HR_max_), the highest observed value was considered.

### Data processing

Elite HRV txt.files were imported into Kubios HRV Software Version 3.5.0 (Biosignal Analysis and Medical Imaging Group, Department of Physics, University of Kuopio, Kuopio, Finland, Tarvainen et al. 2014). Preprocessing settings were set to the default values including the RR detrending method which was kept at “smoothness priors” (Lambda = 500). DFA-alpha1 window width was set to 4 ≤ n ≤ 16 beats (Peng et al. 1995). Artifacts in the RR series were corrected by the Kubios “automatic method” (Lipponen and Tarvainen 2019) and excluded from further analyses when the overall percent artifact exceeded 5% (Rogers et al. 2021c). Options for the time-varying analysis were adjusted to a 2 min window width (Chen et al. 2002) and 5 s grid interval for the moving window. The exported csv.files contained DFA-alpha1 and HR values recalculated every 5 s.

### Threshold determination

VT1, VT2 and HRVT1, HRVT2 were, respectively, determined at the outcomes of HR and VO_2_ by means of regression (VO_2_ over time, DFA-alpha1 over time, DFA-alpha1 over HR). According to the approach of Rogers et al. (2021a, b, c, d, e), oxygen uptake (VO_2_), carbon dioxide (VCO_2_), end-tidal oxygen concentration (PetO_2_), end-tidal carbon dioxide concentration (PetCO_2_) and minute ventilation (VE) have been used for a fourfold determination of VT1 by means of modified V-slope method, ventilatory equivalencies, excess CO_2_ production and PetO_2_ nadir. The first three procedures were based on the recommendations of Gaskill et al. (2001) and the last one was suggested by Binder et al. (2008). VT2 was determined using respiratory compensation point and deflection point of PetCO_2_ (Binder et al. 2008). Time points of VT1 and VT2 occurrence were first set by the investigation leader and were mutually agreed upon by two other investigators. Data points were inserted in the linear regression equation to get the respective VO_2_ values. The associated HR for VT1 and VT2 was extracted from the synchronously running HR recording. HRVT1 and HRVT2 (VO_2_) were delineated on the basis of the DFA-alpha1 over time regression equation. For this purpose, only the subset of data consisting of the rapid near linear decline from values near 1.0 (correlated) to approximately 0.50 (uncorrelated) were considered (Rogers et al. 2021a). In case of values below 0.50 for some participants, the data range was extended up to this minimum (1.0 to the lowest observed value). The time points crossing the DFA-alpha1 values of 0.75 and 0.50 allowed for HRVT VO_2_ determination by use of VO_2_ over time relation. HR at DFA-alpha1 0.75 and 0.50 was obtained by plotting HR on the x-axis and DFA-alpha1 on the y-axis. Artifact percentage for DFA-alpha1 assessment during the linear regression segment is listed in Table 1.

### Statistics

The main variables VT1 VO_2_, VT1 HR, HRVT1 VO_2_, HRVT1 HR, VT2 VO_2_, VT2 HR, HRVT2 VO_2_, HRVT2 HR were statistically analyzed using Microsoft Excel 365 and IBM SPSS Statistics Software Version 27. Descriptive data analysis including calculation of means and standard deviations (SD) was applied. Normal distribution of data was verified by Shapiro–Wilk’s test. The relationship of VT1 and VT2 with HRVT1 and HRVT2 was assessed using intraclass correlation coefficient (ICC_3,1_), linear regression, Pearson’s r correlation coefficient, standard error of estimate (SEE), coefficient of determination (R^2^) and Bland Altman plots with limits of agreement (Bland and Altman 1999). The size of Pearson’s r correlations was evaluated as follows; 0.3 ≤ r < 0.5 low; 0.6 ≤ r < 0.8 moderate and r ≥ 0.8 high (Chan 2003). The paired t test was used for comparison of VT1 vs. HRVT1 and VT2 vs. HRVT2 for both VO_2_ and HR parameters. For all tests, the statistical significance was accepted as p ≤ 0.05. Cohen’s d was used to denote effect sizes (small effect = 0.2, medium effect = 0.5, large effect = 0.8) (Cohen 1988).

## Results

### Data inclusion

From the 31 participants tested, three participants were excluded from analysis due to lack of agreement in the determination of ventilatory thresholds or an untraceable DFA-alpha1 course. A further two were rejected, because they exceeded the overall percent artifact (> 5%).

### Gas exchange testing

Individual gas exchange results are presented in Table 2. VO_2max_ ranged between 27.4 and 44.7 ml/kg/min. VT1 VO_2_ was reached between 62 and 75% of VO_2max_, VT1 HR between 71 and 91% of HR_max_. VT2 showed respective percentages in the range of 75 and 96% of VO_2max_, as well as 88 and 100% of HR_max_.

**Table 2:** Comparison of ventilatory thresholds and heart rate variability thresholds with measures of VO_2_ and HR

Participant	VO_2max_ (ml/kg/min)	HR_max_ (bpm)	VT1 VO_2_ (ml/kg/min)	HRVT1 VO_2_ (ml/kg/min)	VT1 HR (bpm)	HRVT1 HR (bpm)	VT2 VO_2_ (ml/kg/min)	HRVT2 VO_2_ (ml/kg/min)	VT2 HR (bpm)	HRVT2 HR (bpm)	Artifacts (%)
1	35.7	168	23.9	24.6	148	151	28.9	29.1	161	159	0.1
2	36.4	159	25.8	25.8	127	130	31.2	30.3	150	143	0.2
3	33.0	181	23.1	24.5	149	155	30.0	29.5	174	170	0.1
4	39.1	180	27.8	27.8	149	152	31.9	32.0	163	162	0.0
5	44.0	166	32.0	32.5	138	141	41.2	38.6	161	154	0.0
6	36.0	188	26.1	28.4	169	174	31.7	35.0	182	188	0.0
7	36.8	182	24.1	25.2	156	163	29.5	29.8	174	173	0.5
8	34.5	172	23.8	25.6	149	154	30.3	30.2	167	166	0.0
9	30.9	177	23.3	24.5	161	165	29.8	30.1	176	178	0.0
10	36.3	168	25.9	29.6	143	157	31.8	32.7	164	165	0.5
11	30.3	186	21.9	21.3	163	165	28.8	26.4	185	180	0.0
12	35.2	180	25.3	25.5	164	158	32.1	29.8	179	167	0.5
13	38.6	199	27.0	28.4	175	180	34.0	33.5	194	192	0.0
14	30.2	168	19.6	19.1	132	128	25.4	26.2	159	161	0.0
15	38.4	181	27.7	27.5	153	152	32.4	31.1	169	165	1.1
16	37.4	154	25.1	25.2	126	128	32.3	31.6	148	145	0.0
17	34.7	178	23.5	23.5	154	153	30.0	27.0	171	163	0.0
18	44.2	193	27.5	27.5	161	163	34.8	34.9	178	178	0.0
19	42.7	194	26.4	26.8	154	155	32.1	32.0	171	171	0.0
20	27.4	147	19.7	21.1	105	118	23.5	23.5	137	136	1.6
21	32.7	167	22.5	22.2	137	133	29.6	28.5	160	156	0.0
22	35.4	182	24.9	29.0	155	170	31.1	34.1	176	182	0.0
23	36.4	170	26.2	33.5	139	164	32.7	36.6	163	171	0.0
24	44.7	158	29.7	38.0	126	147	38.5	45.4	149	161	0.0
25	41.4	165	28.6	25.8	144	135	35.5	37.4	160	160	0.3
26	32.9	172	23.7	26.1	143	151	28.7	32.9	160	172	0.0
Mean (SD)	36.4 (± 4.5)	174 (± 12.6)	25.2 (± 2.8)	26.5 (± 4.0)	147 (± 15.6)	152 (± 15.5)	31.5 (± 3.6)	31.9 (± 4.5)	167 (± 12.7)	166 (± 13.2)	0.2 (± 0.4)

*VT* ventilatory threshold, *HRVT*  DFA-alpha1 derived threshold, *HR* heart rate, *HR*_*max*_ maximum heart rate, *VO*_*2*_ oxygen uptake, *VO*_*2max*_ maximum oxygen uptake, *SD* standard deviation.

## RR interval quality

The percentage of artifacts was calculated based on the Kubios automatic correction method for each participant’s test data. Since only a portion of the entire exhaustive test was used for the linear interpolation of DFA-alpha1, the artifact percentage listed refers to that section only. Artifact percentage for the linear plotted data series was between 0 and 1.6% (Table 2).

## Comparison of VT1 vs. HRVT1 and VT2 vs. HRVT2

**Fig. 1:**
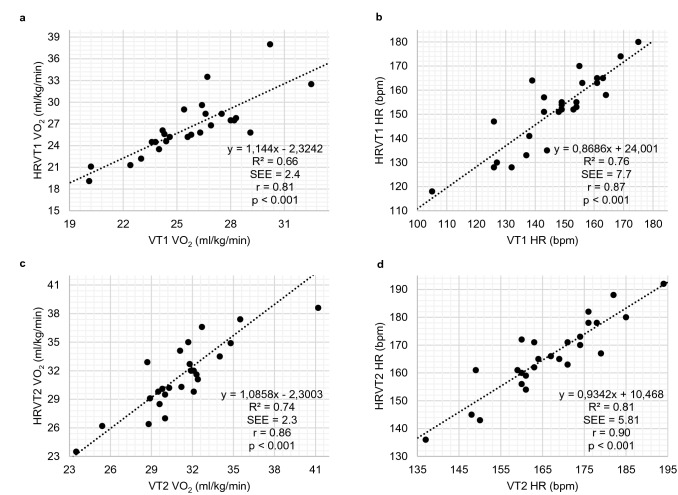
Regression plots for all participants **a** Values of VT1 vs. HRVT1 for VO_2_; **b** Values of VT1 vs. HRVT1 for HR; **c** Values of VT2 vs. HRVT2 for VO_2_; **d** Values of VT2 vs. HRVT2 for HR. SEE, standard error of estimate; R^2^, coefficient of determination

**Fig. 2:**
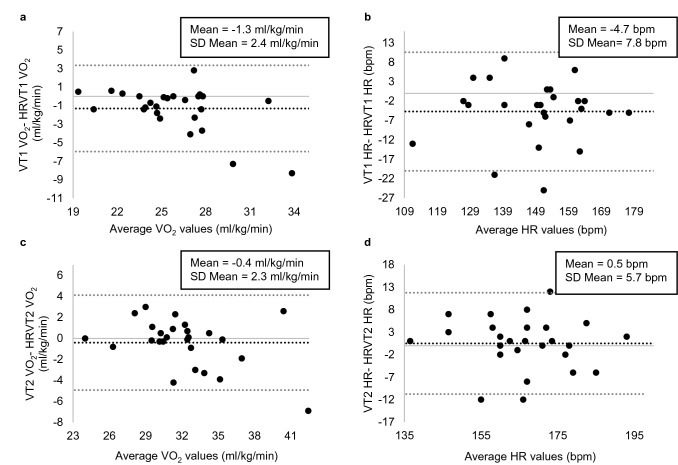
Bland Altman plots of values of **a** VT1 vs. HRVT1 for VO_2_; **b** VT1 vs. HRVT1 for HR; **c** VT2 vs. HRVT2 for VO_2_; **d** VT2 vs. HRVT2 for HR. Center line in each plot represents the mean difference between each paired value, the top and bottom lines are 1.96 standard deviations from the mean difference

The average VT1 VO_2_ and VT2 VO_2_ were 25.2 ml/kg/min (± 2.8) and 31.5 ml/kg/min (± 3.6) compared to 26.5 ml/kg/min (± 4.0) and 31.9 ml/kg/min (± 4.5) obtained by the DFA-alpha1-derived thresholds HRVT1 and HRVT2. The average HR at VT1 VO_2_ was 147 bpm (± 15.6) and 167 bpm (± 12.7) for VT2 VO_2_ compared to 152 bpm (± 15.5) and 166 bpm (± 13.2) obtained by HRVT1 and HRVT2. High correlations could be found between the variables represented in Pearson’s *r* values of 0.81 (VT1 vs. HRVT1, *p* < 0.001, ICC_3,1_ = 0.77) and 0.86 (VT2 vs. HRVT2, *p* < 0.001, ICC_3,1_ = 0.84) for the VO_2_ comparisons and 0.87 (VT1 vs HRVT1, *p* < 0.001, ICC_3,1_ = 0.87) and 0.90 (VT2 vs. HRVT2, *p* < 0.001, ICC_3,1_ = 0.90) for the respective HRs (Fig. 1). Whereas the comparison of VT2 and HRVT2 showed no differences (VO_2_: *p* = 0.385, d = − 0.173; HR: *p* = 0.661, d = 0.086), VT1 vs. HRVT1 revealed significant differences with regard to both metrics (VO_2_: *p* = 0.010, d = − 0.549; HR: *p* = 0.005, d = − 0.601). Bland Altman analysis for VT1 vs. HRVT1 for VO_2_ showed a mean difference of − 1.3 ml/kg/min (± 2.4) with upper and lower limits of 3.3 and − 6.0 ml/kg/min (Fig. 2a). Regarding HR there was a difference of − 4,7 bpm (± 7.8) with upper and lower limits of 10.6 and − 20.0 bpm (Fig. 2b). Bland Altman analysis for VT2 vs. HRVT2 for VO_2_ (Fig. 2c) showed a mean difference of − 0.4 ml/kg/min (± 2.3) with upper and lower limits of 4.1 and − 4.9 ml/kg/min. In addition, a mean deviation of 0.5 bpm (± 5.7) with upper and lower limits of 11.8 and − 10.8 bpm for the respective HR was shown (Fig. 2d).

## Discussion

The present study followed recent published approaches (Rogers et al. 2021a, b, e) to compare workloads at fixed DFA-alpha1 values of 0.75 and 0.50 to the corresponding work rate at VT1 and VT2 during an incremental test. These prior investigations were done in healthy male participants and male participants with cardiac disease. The resulting findings showed strong similarities in terms of VT1 vs. HRVT1 for both demographic groups, whereas VT2 vs. HRVT2 was only studied in the former population, but also showed a good relation in terms of HR (Rogers et al. 2021a, b, e). Since no data were previously available for female participants, the findings of this study are intended to test the utility of DFA-alpha1 for these purposes.

The present analysis showed high correlations for the comparison of VT1 vs. HRVT1. The mean difference for VO_2_ (1.3 ml/kg/min) was nearly in line with the corresponding deviation for men with cardiac disease (1.2 ml/kg/min) (Rogers et al. 2021e) as well as for healthy male participants (0.3 ml/kg/min) (Rogers et al. 2021a). The upper and lower LoA for the comparison of VT1 vs. HRVT1 in the present study showed a wider range for both VO_2_ and HR similar to the group of participants with cardiac disease. However, this difference was rated as minimal from a clinical and practical standpoint. With regard to the VT2 vs. HRVT2 comparison, all analyses confirmed strong agreement in terms of VO_2_ and HR. With a Pearson’s correlation coefficient of 0.90 for the HR comparison, it was even higher than in the study by Rogers et al. (2021b), which yielded a coefficient of 0.78.

Potential factors for DFA-alpha1 bias include artifact correction, recording device type (Rogers et al. 2021c), unsuitable detrending (Voss et al. 2015), fatigue, and stress (Rogers et al. 2021d). Although artifact correction can induce a proportional bias resulting in DFA-alpha1 to decline relatively later in the exercise ramp (Rogers et al. 2021c), this does not apply to the present data since artifact percentage was very low (0.2%). With respect to device induced bias, it has been shown that the H7 Polar chest strap induced an early drop in DFA-alpha1 during incremental testing (Rogers et al. 2021c), not a late one as in our study. Proper detrending was ensured using Kubios HRV software (Tarvainen et al. 2014) mirroring the methods of Rogers et al. (2021a). Finally, fatigue can also cause a premature decline in DFA-alpha1 (Rogers et al. 2021d), but the participants in the present study were well rested.

It seems reasonable that the observed delayed decline and wider LoA of DFA-alpha1 at HRVT1 during incremental ramp testing in many participants could be due to sex specific hormonal differences. It is known that estrogen enhances parasympathetic control of the heart which is why premenopausal women experience higher vagal activity compared to males and postmenopausal women (Adjei et al. 2018). Furthermore, it has been shown in previous studies that the balance of ovarian hormones reflected in the menstrual cycle might play a significant role on the regulation of autonomic tone (Yildirir et al. 2001; Bai et al. 2009). There is a plethora of data on linear HRV indexes with respect to the female cycle, the vast majority of which demonstrate a decrease of the vagal dominance on the heart from the follicular to the luteal cycle phase (von Holzen et al. 2016). To date, there is very limited data on non-linear HRV properties and no data regarding intensity thresholds. Although the few existing studies also point to a loss of complexity in the late cycle phase, the results must be treated with caution due to methodological limitations like low data sampling rates, processing without Kubios HRV software or different measurement time points of assessing HRV (Bai et al. 2009; Rawal 2014).

The present study is the first to examine DFA-alpha1 behavior during exercise in females. Based on the present findings, it is possible that the HRVT1 slightly shifts with the fluctuations in ovarian hormones across the menstrual cycle or with female hormone status in general. Definitive evaluation of this situation is problematic since every woman possesses an individual endogenous hormonal profile which is further affected by exogenous factors such as physical activity and exercise habits, performance level, diet habits, hormonal contraceptives or hormone replacement therapy (Elliott-Sale et al. 2021). This can be best seen in the partially contradictory results on the influence of premenstrual syndrome (PMS) or premenstrual dysphoric disorder (PMDD) on HRV (von Holzen et al. 2016). The delay in DFA-alpha1 decline during the exercise ramp resulting in slight HRVT1 bias may also relate to the observation that women are more resistant to neuromuscular fatigue during endurance exercise (Temesi et al. 2015). It is plausible that reduction or delay in the onset of neuromuscular fatigue can affect DFA-alpha1 kinetics (Rogers et al. 2021d).

Interestingly, despite the issues surrounding agreement between HRVT1 and VT1 in the female participants, the conformity between HRVT2 and VT2 was similar to that reported in men (Rogers et al. 2021b). A possible reason revolves around the underlying nature of the HRVT2 which corresponds to a loss of physiologic sustainability and organismic destabilization (Rogers et al. 2021b), therefore, more resistant to hormonal changes.

Overall, the agreements obtained in this study seem comparable to those of conventional surrogate markers. Denis et al. (2021), for example, showed similar differences in the thresholds between blood lactate and gas exchange during a stage protocol in 14 males. It is also important to mention that the agreement of physiological markers for threshold determination may also vary due to changes in the work rate increase during incremental tests (Hughson and Green 1982), pedaling frequency (Hughes et al. 1982), inter- and intra-rater agreement (Gladden et al. 1985), glycogen depletion (Hughes et al. 1982) and determination methods used (Gaskill et al. 2001). Additionally, the mean differences presented in this study are of similar magnitudes as Functional Threshold Power (FTP) vs. Maximal Lactate Steady State (MLSS) (Klitzke Borszcz et al. 2019) or deoxygenated hemoglobin breakpoints vs. MLSS (Bellotti et al. 2013) and thus show similar practical utility as a non-invasive marker for an anaerobic threshold.

From a practical standpoint, the cutoff values of 0.75 and 0.50 seem to be well-suited markers for zone 1 to 2 and 2 to 3 transition (in a three zone model) in most of the women observed. This method of estimation of physiological breakpoints demonstrates the usability of DFA-alpha1 as a marker of organismic demands and internal load. Unlike other physiological and subsystemic indicators (like gas exchange or blood lactate concentration indicators), the correlation properties of HRV offer a more integrated perspective of autonomic nervous system regulation (Gronwald et al. 2020b). These regulation patterns could be seen as an outcome of the concepts underlying network physiology (Balagué et al. 2020) which embody the complex dynamic interplay of electro-physiological, hemodynamic, and humoral variables, along with the effects of the autonomic and central nervous system regulation (Gronwald et al. 2020b). When intensity reaches a moderate to high level, a disintegrating process is initiated, while culminating into progressive segregation of subsystems and mechanization (e.g. performance attractor) of the entire system (Gronwald et al. 2020a, b).

## Limitations and future directions

Future work should further investigate possible factors influencing the course of DFA-alpha1 in women. Due to the varied endogenous hormone profiles influenced by external factors like hormonal contraceptives, diet or exercise habits, specific considerations should be made regarding participant selection criteria and adaptations for experimental design according to the recommendations of Elliott-Sale et al. (2021). This would demand a recruitment based on pre-defined, standardized criteria as well as methodological decisions in terms of the timing of testing throughout the menstrual cycle (Elliott-Sale et al. 2021). In addition, exploration of endurance exercise induced neuromuscular fatigue between male and females in association with DFA-alpha1 behavior may yield interesting insights into alteration of gender specific autonomic balance during these kinds of sporting activities.

Since the the recording methodologies prescribed involve a post-session data interpretation, it would be recommendable to follow-up on the work of Gronwald et al. (2021) who illustrated the potential of real-time DFA-alpha1 and intensity distribution monitoring during endurance exercise. This could serve as a milestone for a further development of apps or even smart devices (e.g. smart watches) able to display this metric and thus give immediate feedback of systemic internal load during exercise (Rogers and Gronwald 2022).

## Conclusion

Non-linear heart rate variability analysis during a cycle ergometer ramp demonstrated good agreement between the heart rate and oxygen uptake at first and second ventilatory threshold and the metrics related with DFA-alpha1 cutoff values of 0.75 and 0.50 in a population of randomly selected women. Although promising, additional study is recommended in terms of validating commercially available recording methods, the verification of other exercise protocols and sporting activities. Furthermore, it would be interesting to investigate DFA-alpha1 behavior in women in the context of their menstrual cycle, and pre vs post menopause status. If investigations into these matters confirm the present results, it may be feasible to extend traditional approaches for intensity distribution assessment (e.g. based on lactate or gas exchange measurement) with real-time calculation of DFA-alpha1 via a wearable device during endurance exercise.

## Data Availability

The data sets used in this study are available from the corresponding author on reasonable request.

## Abbreviations

AT: Aerobic threshold
DFA: Detrended fluctuation analysis
ECG: Electrocardiogram
HR: Heart rate
HR_max_: Maximum heart rate
HRV: Heart rate variability
HRVT: DFA-alpha1 derived threshold
PetCO_2_: End-tidal carbon dioxide concentration
PetO_2_: End-tidal oxygen concentration
RPE: Rating of perceived exertion
SDNN: Total variability as the standard deviation of all normal RR-intervals
VCO_2_: Carbon dioxide
VE: Minute ventilation
VO_2_: Oxygen uptake
VO_2max_: Maximum oxygen uptake
VT: Ventilatory threshold
